# Efficacy of the gluten free diet in the management of functional gastrointestinal disorders: a systematic review on behalf of the Italian Society of Paediatrics

**DOI:** 10.1186/s13052-019-0606-1

**Published:** 2019-01-11

**Authors:** Elena Scarpato, Renata Auricchio, Francesca Penagini, Angelo Campanozzi, Gian Vincenzo Zuccotti, Riccardo Troncone

**Affiliations:** 1 Department of Translational Medical Sciences – Section of Paediatrics, University of Naples Federico II, via Pansini 5, 80131 Naples, Italy; 2 Department of Pediatrics, University of Milan, V. Buzzi Children’s Hospital, via Castelvetro 32, 20154 Milan, Italy; 3 Pediatrics, Department of Medical and Surgical Sciences, University of Foggia, via Luigi Pinto 1, 71100 Foggia, Italy

**Keywords:** Irritable bowel syndrome, Elimination diet, Abdominal pain, Gastrointestinal symptoms

## Abstract

**Background:**

Functional gastrointestinal disorders (FGIDs) are characterized by chronic/recurrent gastrointestinal symptoms not related to organic disorders. Due to the limited treatment options and to the perception of subjects with FGIDs suffering from a food intolerance, in recent years there has been an increase in the self-prescription of elimination diets, especially gluten free diet (GFD), for the treatment of these disorders. For this reason, we decided to perform this systematic review with the aim to evaluate the available evidence on the effects of a GFD on gastrointestinal symptoms, in subjects with FGIDs.

**Methods:**

Cochrane Library and MEDLINE (via PubMed) databases were searched, from inception to March 2018, using the MeSH terms “functional gastrointestinal disorder OR irritable bowel syndrome AND gluten”. We included all the clinical trials published in English and evaluating the effects of a GFD in subjects with FGIDs diagnosed according to the Rome II, III, and IV criteria.

**Results:**

Eleven trials were eligible (3 prospective trials, 8 single or double-blind placebo-controlled trials), with 10/11 trials including adult subjects with irritable bowel syndrome (IBS) or FGIDs. Most of the prospective studies found an effect of GFD on gastrointestinal symptoms control. Nevertheless, 1 trial failed to find an association between gluten and GI symptoms when FODMAPs (fermentable oligosaccharides, disaccharides, monosaccharides and polyols) content was simultaneously reduced in the diet, and 2 trials reported a worsening of symptoms during placebo administration. The results of the different trials are difficult to compare due to discrepancies in the study protocols regarding the amount and type of gluten administered, the duration of the gluten challenge, the type of placebo used, and the duration of the challenge itself.

**Conclusions:**

According to our results, gluten may contribute to the occurrence of gastrointestinal symptoms in patients with FGIDs, particularly in those with IBS. Nevertheless, the results of the currently available trials are difficult to compare due to the lack of standardization in the study designs. For this reason, it is still not possible to recommend the use of the GFD in the routine management of FGIDs.

## Background

Functional gastrointestinal disorders (FGIDs) define a group of chronic or recurrent gastrointestinal symptoms, not explained by known organic disorders [1]. The pathogenesis of FGIDs is multifactorial, with several mechanisms contributing to their onset, including visceral hyperalgesia, gastrointestinal (GI) motility disturbances, environment, genetic factors, brain-gut axis alterations, and psychosocial factors. FGIDs are extremely frequent, with a prevalence of 23% in the paediatric population from the European-Mediterranean Area [2]. Due to the lack of specific markers, they are currently diagnosed according to the Rome criteria that provide a standardized definition and classification for FGIDs. Since the first meeting of the paediatric working team in 1997 [1], the Rome criteria have been regularly updated [3, 4]. The clinical advantage of using the Rome criteria is that they allow a “positive” approach to the patient, avoiding unnecessary tests to rule out an organic cause, with a beneficial effect on both patient’s health and healthcare costs. In fact, FGIDs are associated with significant morbidity and high costs, with influence on socialization, school absenteeism, and long-term psychological implications, with quality of life scores comparable to that of subjects with inflammatory bowel disease [5], and a significant proportion of these patients that continues to have symptoms into adulthood [6, 7].

One of the major issues in the clinical management of FGIDs is the treatment. Current pharmacological therapies are mainly targeted at managing the predominant symptom, with an action that could be either peripheral (e.g. antispasmodic drugs) or central (e.g. antidepressants). However, the long-term effectiveness of these approaches is extremely variable. Some evidence exists on the efficacy of centrally directed treatments, such as cognitive behavioural therapy and hypnotherapy; nevertheless, these interventions are time-consuming and costly, and are not available in the majority of clinical settings. This makes the treatment of FGIDs a great unmet need in modern paediatric gastroenterological practice. Because of these limited treatment options, in recent years there has been an increase in the self-prescription of elimination diets in subjects affected by FGIDs, especially in patients with irritable bowel syndrome (IBS). This is mainly due to the fact that, in subjects with IBS, the perception of suffering from a food intolerance is more common than in the general population, with up to 60% of the patients referring GI symptoms between 15 min and 3 h after the intake of specific foods [8]. The mechanisms by which food can cause GI symptoms are various, including immune system stimulation and activation of intestinal mechanoreceptors. The hypothesis of an activation of the immune system is supported by the evidence of an intense mast cell infiltration in intestinal biopsies of subjects with IBS [9]. An explanation for this low-grade inflammation is that specific food antigens could overcome the intestinal barrier and stimulate an immune response, resulting in mast cell infiltration, release of inflammatory mediators and onset of GI symptoms. Regarding the pathogenetic role of specific mechanoreceptors, this is supported by the evidence that the interaction between dietary factors and intestinal microbiota in the gut lumen causes fermentation, gas production and intestinal distension that, in the presence of visceral hyperalgesia and alterations of the GI motility, could be responsible for the onset of pain and bowel habit changes [10].

Non-coeliac gluten sensitivity (NCGS) is characterized by intestinal (e.g. bloating and abdominal pain) and extra-intestinal symptoms (e.g. headache, anxiety, fibromyalgia-like syndrome and skin rash) subsequent to the ingestion of gluten, in subjects without coeliac disease or wheat allergy [11].

NCGS can occur at any age. However, it is rare during childhood, arising more frequently in adulthood, with a peak in the fourth decade of life [12]. IBS-like complaints are often part of the clinical picture of NCGS, with NCGS individuals often fulfilling the Rome criteria for IBS, with a frequent overlap between these two conditions. The main difference is that subjects with NCGS tend to clearly identify gluten as the culprit for the occurrence of GI symptoms, while IBS patients do not directly correlate gluten to the symptomatology [13]. Still, wheat is one of the foods more frequently associated with the onset of GI symptoms in subjects with IBS.

On the other hand, it is still not clear which component of wheat is responsible for the clinical effects: proteins (especially gluten), or carbohydrates (especially, fermentable oligo-di-mono-saccharides and polyols – FODMAPs) [10]. However, regardless of which component is responsible there is an agreement that wheat elimination can improve GI symptoms in a subgroup of patients with IBS, that can be defined as affected by wheat-sensitive IBS [13].

The aim of the present systematic review is to evaluate the available evidence on the effects of a gluten free diet (GFD) on GI symptoms, in subjects with FGIDs.

## Methods

### Search strategy and selection criteria

A computerized literature search was conducted from inception to March 2018 through MEDLINE (via PubMed) using the MeSH terms “functional gastrointestinal disorder OR irritable bowel syndrome AND gluten”, and through the Cochrane Library, using the MeSH term “functional gastrointestinal disorder” and the subheading “diet therapy”. Trials published in English and evaluating the effects of a GFD in subjects with FGIDs diagnosed according to the Rome II, III, and IV criteria were included. Trials evaluating subjects affected by coeliac disease, wheat allergy, or other gluten-related disorders (gluten sensitivity) were excluded. Due to the paucity of studies conducted on paediatric subjects, we decided to include also studies enrolling adult subjects. Reference citations from all the included studies were searched to add further appropriate publications. Duplicate publications were excluded.

### Data extraction and quality assessment

Title and abstract of the included studies were independently screened by two authors (RA and ES). Full texts were obtained only for studies meeting the inclusion criteria. The eligibility of full text articles was independently assessed by two authors (RA and ES). Disagreement on study eligibility were discussed and resolved with a third author (RT). Data from the included studies was independently extracted by two authors (AC and FP) using a predefined scheme. Any discrepancy between the two sets of data extracted was discussed with a third author (ES). Two authors (AC and FP) independently evaluated the risk of bias of the selected studies using the Cochrane risk of bias tool [14] including the following domains: random sequence generation (selection bias), blinding of participants and personnel (performance bias), allocation concealment (selection bias), blinding of outcome assessment (detection bias), incomplete outcome data (attrition bias) and selective reporting (reporting bias), while as “other bias” we included the lack of a control group (design bias). For each outcome, the risk of bias was defined as “low”, “high”, or “unclear” (Table 1).

**Table 1:** Risk of bias of the included studies, rated according to the Cochrane risk of bias tool, as high, unclear or low

Reference	Random sequence generation (Selection bias)	Allocation concealment (Selection bias)	Blinding of participant and personnel (Performance bias)	Blinding of outcome assessment (Detection bias)	Incomplete outcome data (Attrition bias)	Selective reporting (Reporting bias)	Choice of control groups (Bias in design)
Adult studies							
Wahnschaffe et al. [14]	High risk	Unclear risk	High risk	High risk	Low risk	Low risk	Low risk
Biesiekierski et al. [15]	Low risk	Low risk	Low risk	Low risk	High risk	Low risk	High risk
Vazquez-Roche et al. [16]	Low risk	High risk	High risk	High risk	Low risk	High risk	High risk
Biesiekierski et al. [17]	Low risk	High risk	Low risk	Low risk	High risk	Low risk	High risk
Aziz et al. [18]	High risk	High risk	High risk	High risk	Low risk	Low risk	High risk
Barmeyer et al. [19]	High risk	Low risk	High risk	High risk	Low risk	Low risk	High risk
Carroccio et al. [20]	High risk	Low risk	Low risk	Low risk	Low risk	Low risk	Low risk
Shahbazkhani et al. [21]	Low risk	Low risk	Low risk	Low risk	Low risk	Low risk	Low risk
Elli et al. [22]	Low risk	Low risk	Low risk	Low risk	Low risk	Low risk	High risk
Zanwar et al. [23]	Low risk	Low risk	Low risk	Low risk	High risk	Low risk	Unclear risk
Paediatric studies							
Francavilla et al. [25]	Low risk	Low risk	Low risk	Unclear	Unclear	Low risk	Low risk

## Results

**Fig. 1:**
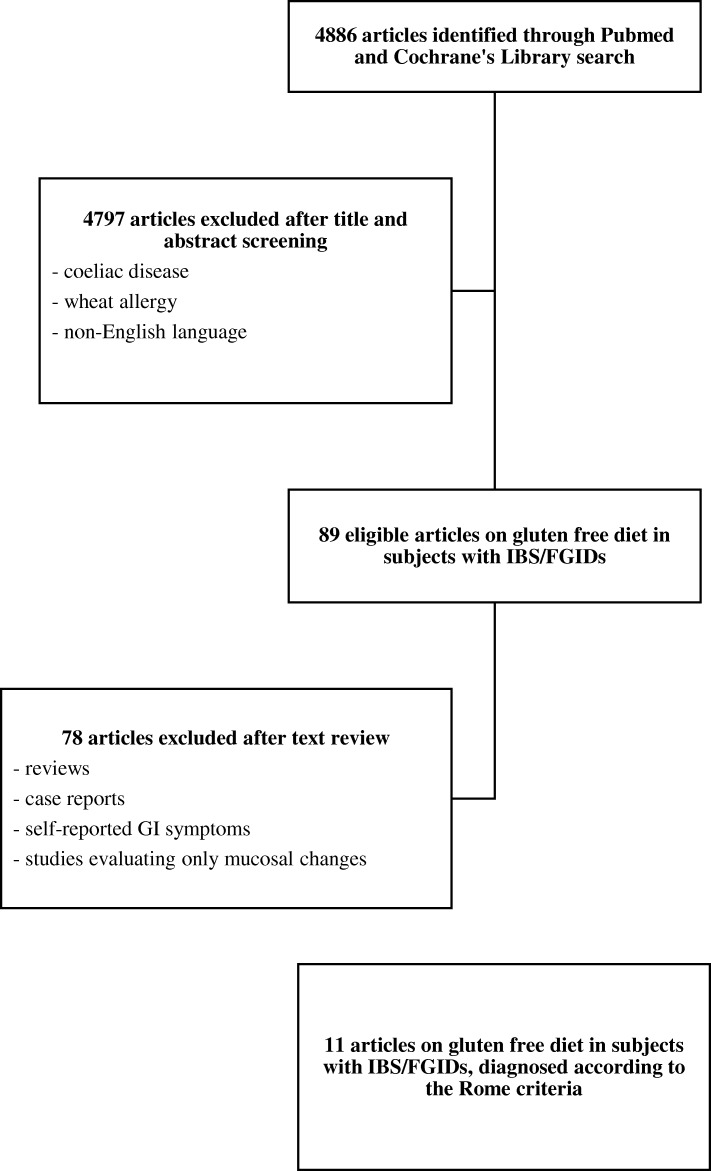
Flow chart of the literature search

The selection process is described in Fig. 1. Starting from the 4886 articles identified in our search, we finally selected 11 clinical trials evaluating the effect of a GFD on GI symptoms, in subjects with FGIDs. All the studies were published in the past 10 years, included subjects with IBS or FGIDs diagnosed according to the Rome criteria, and excluded the presence of coeliac disease. However, 5 studies did not report the methods used to rule out wheat allergy [15–19]. Regarding the study design, 1 study was a single-blind, randomized controlled trial [17], 3 were prospective studies evaluating only the clinical response to a GFD, without performing a gluten challenge [15, 19, 20], while the remaining 7 studies were double-blind placebo-controlled trials (DBPCT) [16, 18, 21–25], and 4 of them also had a crossover design [18, 21, 23, 25].

Table 2 summarizes data regarding the study design, inclusion criteria, outcomes, results and risk of bias of the 11 studies included.

**Table 2:** Study design, outcomes, and results of 11 trials evaluating gluten free diet in subjects with FGIDs

Author, Year, Country [ref.]	Type of Study, study population, intervention, evaluation scale	Inclusion Criteria	Outcomes/Endpoints	Results
Adult studies				
Wahnschaffe et al. 2007, Germany [15]	-Prospective −41 IBS-diarrhea (30–67 years); 102 controls (27–64 years) -GFD: 6 months − 5 GI symptoms graded with a 5-point Likert scale.	-IBS-diarrhea (Rome II)	-During the GFD, resolution of diarrhea and decrease in GI symptoms score below the mean + 2 standard deviations score of healthy controls,	- In 41 IBS-diarrhea patients the GI symptom score decreased from 14.5 ± 0.9 to 10.1 ± 0.9 (*P* < .01) - Decrease in stool frequency from 4.1 ± 0.2 to 2.0 ± 0.3 bowel movements/day (*P* < .01). - 15/41 (37%) had formed stools and normal frequency; 12/41 (29%) both GI symptoms and stool habits returned to normal.
Biesiekierski et al. 2011, Australia [16]	-Double blind, placebo-controlled − 34 subjects (29–59 years) -GFD: at least 6 weeks before screening -Challenge: 6 weeks -Gluten containing muffin and bread (16 g/day) vs gluten free muffin and bread - Symptom questionnaire with 100-mm VAS.	-IBS (Rome III)	- Efficacy of the diet (gluten containing or GFD) in GI symptoms control. - Change in overall and individual GI symptoms.	- For more than half of the study duration, in 13/19 (68%) subjects in the gluten group the symptoms were not adequately controlled vs 6/15 (40%) in the placebo group, (*P* = .001) - The severity scores for pain, satisfaction with stool consistency, and tiredness were significantly higher for the gluten containing diet. - All patients experienced a worsening of symptoms in response to gluten by the first week of re-challenge, in contrast to the placebo group in which symptoms occurred more slowly and less severely.
Carroccio et al. 2012, Italy [21]	-Double blind, placebo-controlled, crossover − 276 NCWS, 50 IBS, 100 CD (> 18 years) -Challenge: 2 weeks; Washout: 1 week -GFD: 4 weeks prior the challenge (also exclusion of cow’s milk, eggs, tomato, and chocolate) -Capsules containing wheat vs xylose -Symptoms with 100-mm VAS	-NCWS with IBS (Rome II) -IBS, no WS (negative at challenge) -CD	- Resolution of symptoms on elimination diets and recurrence after DBPC challenge.	- In the 276 NCWS patients the overall and the single symptom score for bloating, abdominal pain, and changes in stool consistency was significantly higher than baseline from the first week on the wheat containing diet (*P* < .0001) On placebo, there was no significant variation in score over baseline.
Vazquez-Roche et al. 2013, USA [17]	- Single-blind, randomized, controlled − 45 subjects: 22 gluten group (43.4 ± 2.7 years); 23 gluten free group (41.8 ± 2.5 years) -Challenge: 4 weeks -Gluten vs rice starch -Daily bowel pattern diary	-IBS-diarrhea (Rome II)	-Effect of a gluten containing diet vs a GFD on bowel function	- Statistically significant decrease in the stool frequency of subjects on a GFD compared to a gluten containing diet (*P* = .04). - No effect on stool form.
Biesiekierski et al. 2013, Australia [18]	-Double blind, randomized, crossover - 37 subjects (range 24–61 years) - 2 weeks run-in low FODMAPs diet, then GFD low in FODMAPs throughout. During the re-challenge, GFD, low FODMAPs, dairy free and low food chemicals. -Trial: 7 days; Washout: at least 2 weeks - Re-challenge: 3 days; Washout: at least 3 days -Trial: high gluten (16 g/day) vs low gluten (2 g gluten + 14 g whey/day) vs control (16 g whey/day) -Re-challenge: gluten (16 g/d) vs whey (16 g/d) vs placebo (no protein) -Symptoms with VAS	-IBS (Rome III)	-Change in overall symptoms score -Change in overall symptoms (> 20 mm VAS) from the run-in period (low FODMAPs) to the end of the study (gluten vs placebo).	- Reduced GI symptoms and fatigue in the run-in period (low FODMAPs diet) (*P* < .0001). - 22% had a mean VAS-improvement for overall abdominal symptoms of > 20 mm during the low FODMAPs diet. - During the re-challenge, no differences across the dietary treatment arms compared to the baseline (*P* > .209). -Specific and reproducible induction of symptoms with gluten was not demonstrated.
Shahbazkhani et al. 2015, Iran [22]	-Double blind, placebo-controlled − 72 subjects: 35 gluten group (mean age 44.5 ± 10 years); 37 placebo group (mean age 43.2 ± 17 years) -Trial: 6 weeks -Gluten meal 100 g (free of FODMAPs and proteins, including 52% gluten or gliadin, 2.3% non-gluten and 27.7 g glucose) vs Placebo meal 100 g (gluten free powder, rice flour, corn starch and glucose) -Symptoms with a VAS (0–10)	-IBS (Rome III)	-Change in overall symptoms score	- Significant differences between the gluten-containing group and the placebo group symptoms control (*P* < .001). - After 6 weeks, symptoms were adequately controlled only in 9/35 (25.7%) of the gluten group vs 31/37 (83.8%) of the placebo group (*P* < .001).
Aziz et al. 2016, UK [19]	- Prospective - 41 subjects (40.4 ± 14.9 years) - GFD: 6 weeks - Symptoms with IBS-SSS	- IBS-diarrhea (Rome III)	- Change in IBS symptom severity score. Clinical response with 50 points decrease.	- In 29/41 subjects (71%)., GFD reduced IBS-SSS of at least 50 points No differences between HLA-DQ groups. - The mean total IBS-SSS decreased from 286 to 131 points after 6 weeks on GFD (*P* < .001).
Elli et al. 2016, Italy [23]	-Double blind, placebo-controlled gluten challenge, crossover − 98 subjects (mean age 38.9 ± 12.7 years) -Trial: 1 week; Washout: 1 week -GFD: 3 weeks before the trial - Capsules containing gluten 5.6 g/day vs rice starch -Symptoms with VAS 10 cm	-Functional GI symptoms (Rome III)	-Resolution of symptoms on elimination diet and recurrence after DBPC challenge	- More pronounced worsening of well-being with gluten than with placebo assumption (*P* = .05). - 28/98 (28.5%) relapsed during the gluten challenge, with 14/28 (50%) relapsing also during the placebo challenge.
Zanwar et al. 2016, India [24]	-Double blind, placebo-controlled − 60 subjects: 30 gluten group (18–60 years); 30 placebo group (18–56 years) -Trial: 4 weeks - 2 slices of gluten containing bread vs 2 slices of gluten-free bread -Symptoms with 100-mm VAS	-IBS (Rome III)	-Change in overall symptoms score	- Worsening of overall symptoms in 55% of the gluten group vs 33% of the placebo group (*P* < .05). - Median VAS scores for abdominal pain, bloating, and tiredness significantly higher in the gluten group compared to the placebo group.
Barmeyer et al. 2017, Germany [20]	- Prospective - 35 subjects - GFD: 4 months - Weekly Subject’s Global Assessment (SGA) - Symptoms with IBS-SSS	- IBS non-constipated (Rome III)	- Relief of IBS symptoms evaluated with the SGA on at least 75% of weeks over a 4-months period of GFD. - Clinical response with 50 points decrease.	- Considerably relief showed for 12/35 (34%) subjects.
Paediatric studies				
Francavilla et al. 2018, Italy [25]	-Double blind, placebo-controlled, crossover - 28 children (11.4 ± 4.3 years) - 2 weeks run-in open GFD - Trial: 2 weeks; Washout: 1 week; -Sachets of gluten (10 g/day) or placebo (rice starch) -Daily symptoms with VAS 10 cm - Weekly symptoms with IBS-SSS	-Functional GI symptoms (Rome III)	- Decrease of at least 30% of the global VAS from the gluten challenge to the placebo challenge - Prevalence of NCGS	- Decrease of all clinical scores (VAS, IBS-SSS) during the open GFD. - 11/28 (39.2%) patients had a global VAS variation > 30% between the gluten challenge group and the placebo challenge group. - No difference in the severity of the global VAS score during the gluten vs placebo challenge. - 4/28 (14.3%) large increase of symptoms with placebo

FGIDs: functional gastrointestinal disorders; *GFD* gluten free diet; *GI* gastrointestinal; *IBS* irritable bowel syndrome; *NCWS* non-coeliac wheat sensitivity; *WS* wheat sensitivity; *NCGS* non-coeliac wheat sensitivity; *VAS* visual analog scale; *IBS-SSS* irritable bowel syndrome-symptom severity score; *CD* coeliac disease; *FODMAPs* fermentable oligo-di-mono-saccharides and polyols; *PBMCs* peripheral blood mononuclear cells; *SGA* subject’s global assessment

### Prospective studies evaluating the effect of a gluten free diet on GI symptoms

Three of the studies are prospective trials evaluating the effect of a GFD on GI symptoms, without performing a gluten challenge. Wahnschaffe et al. [15] included 41 adult subjects with IBS to follow a GFD for 6 months. They evaluated the HLA-DQ2 status and the effect of the diet on stool frequency, GI symptoms, anti-gliadin IgG and tissue-transglutaminase antibodies. They also recruited 102 healthy volunteers as a control group for the GI symptoms. After 6 months of GFD, 41 IBS patients showed a significant decrease in stool frequency and GI symptom score, that in 20/41 (49%) improved to scores within the normal range. They also found that increased anti-gliadin (AGA) IgG and/or tissue-transglutaminase antibodies were more frequent in IBS patients who expressed the HLA-DQ2 compared to HLA-DQ2–negative IBS patients (*P* < .05) and that a normal GI symptom score after GFD was achieved more frequently in those patients who were HLA-DQ2-positive and/or had celiac disease–associated IgG antibodies. Finally, diarrhea resolved more frequently in HLADQ2–positive patients with celiac disease–associated IgG antibodies. According to their data, serum AGA IgG or tissue-transglutaminase antibodies in combination with HLA-DQ2 expression could be useful to identify a subgroup of patients with IBS more likely to respond to a GFD. These findings are in contrast with the results of the second prospective trial, performed in 2016 by Aziz et al. [19], who evaluated the HLA DQ2/8 status and the GI symptom score of 41 adults with IBS-diarrhea who were asked to follow a GFD for 6 weeks. At the end of the GFD, 29/41 (71%) subjects showed a significant reduction of the IBS-Symptom Severity Score (IBS-SSS), irrespective of the HLA-DQ2/8 status. Their findings are similar to the ones of Barmeyer et al. [20] that enrolled 35 adults with IBS to evaluate overall well-being and GI symptoms response to a 4-months GFD, in relation to HLA DQ2/8 status. At the end of the 4-months follow-up, 12/35 (34%) subjects had a significant improvement of the subject’s global assessment and were classified as having a wheat sensitivity, with 3/12 (25%) that reached a complete relief. However, no association was found between HLA-DQ2/8 expression and wheat sensitivity. Data from all the trials are summarized in Table 2.

### Single and double blind, placebo controlled trials evaluating the effect of gluten on GI symptoms

#### Adult studies

Seven studies are controlled trials, with a single or a double-blind design, evaluating the effect of gluten on GI symptoms, in adults. In 2011 Biesiekierski et al. [16] performed a DBPCT in 34 adult patients with IBS. According to the study protocol, all subjects had to follow a GFD throughout the study period and were asked to consume, every day for 6 weeks, one muffin and two slices of bread with or without gluten. The primary outcome was the symptom control, assessed with a specific question, while the secondary outcome was the change in overall and individual GI symptoms (bloating, abdominal pain, satisfaction with stool consistency, nausea and tiredness) assessed with a visual analog scale (VAS). They also evaluated the HLA-DQ2/8 status of the patients included. At the end of the study, 13/19 (68%) patients in the gluten group reported poor symptom control compared to 6/15 (40%) in the placebo group (*P* < .001). Moreover, patients in the gluten group had greater changes in overall symptoms from baseline to the end of week 1, and higher severity scores over the entire study period than those in the placebo group. The symptomatic response to gluten was not influenced by HLA status. However, in 2013 the same research group performed a new DBPCT on 37 adult subjects with IBS to evaluate the specific effects of gluten after dietary reduction of FODMAPs [18]. All the patients followed a low FODMAPs diet for a 2-weeks run-in period and were then asked to continue a GFD low in FODMAPs for the entire study period. All patients received 1 of 3 diets (high or low gluten, or placebo) for 1 week and, after a washout period, had to crossover to another intervention. A re-challenge was also performed, during which all subjects received 1 of 3 diets (gluten, whey, or placebo) for 3 days, followed by a washout before crossing over to the next intervention. Symptom score was evaluated on a VAS. Considering the 7-days trial, the average of symptoms improved already during the second week of the low FODMAPs diet, with 8/37 (22%) subjects showing a significant improvement compared to baseline, and only 6/37 (16%) having an increase in overall symptoms during the high-gluten diet. It was not possible to observe a dose effect of gluten, with a symptomatic response to gluten identified in only 3/37 (8%) and with 11/37 (30%) showing a positive response in the placebo arm. During the 3-days re-challenge, changes in individual symptoms were similar across the 3 dietary interventions (all *P* > .209), with gluten specificity that was not reproduced in any subject. Another trial published in 2012 [21] is a retrospective chart review that included 276 subjects with an IBS-like presentation and non-coeliac wheat sensitivity (NCWS) diagnosed with a DBPC challenge. Notably, the original cohort included 920 IBS patients who underwent an elimination diet followed by a DBPC re-challenge, finding that 276/920 (30%) had symptoms during gluten ingestion. Subjects with NCWS were divided in 2 groups: 70 with wheat sensitivity (WS) alone, and 206 with WS and multiple food hypersensitivity (MFH). HLA-DQ status and serum AGA were also evaluated. All subjects had to follow a 4-weeks diet with the exclusion of cow ‘s milk, wheat, eggs, tomato, and chocolate. Considering wheat reintroduction, the DBPC re-challenge was performed using wheat or xylose for 2 weeks, followed by a 1-week washout before crossing over to the other treatment. GI symptoms were evaluated on a VAS. Median time for a clinical reaction after gluten ingestion was 3 days for WS-alone subjects and 2.5 days for those with WS and multiple food hypersensitivity. Moreover, 10 patients with WS alone and 32 with WS-MFH did not complete the challenge due to the severity of symptoms during wheat assumption. In all subjects, starting from 1 week after reintroduction of wheat, the VAS for GI symptoms was significantly higher compared to baseline (*P* < .0001). In addition, subjects with WS had higher rates of positive AGA IgA and IgG compared to IBS subjects without WS (*P* = .0001 and P = .0001, respectively), and subjects with WS alone had a higher frequency of HLA DQ2 or DQ8 positivity compared to subjects with WS-MFH (P = .0001). The only single-blind trial included is a study from Vazquez-Roche et al. [17], in which 45 subjects with IBS-D were enrolled to follow a 4-weeks gluten containing/free diet. The primary outcome was to evaluate the effect on bowel frequency and form. They also performed HLA genotyping of DQ alleles, measurement of gastric emptying, small bowel and colonic transit, permeability and morphology, quantitation of tight junction proteins, and evaluation of proliferative responses of peripheral blood mononuclear cells’ (PBMCs) cytokines to gluten and rice. Their data showed a significant decrease in stool frequency during the GFD compared to the gluten containing diet (*P* = .04), with a greater effect in HLA-DQ2 or DQ8 positive subjects. No diet effect on stool form, gastric emptying, colonic transit, mucosal morphology, and PBMCs proliferation was found. On the contrary, they reported a change in occludin expression in the small bowel mucosa of HLA-DQ2 or DQ8 subjects associated to the GFD, an increased small bowel permeability during the gluten containing diet, and a greater induction of IL-10 by PBMCs when stimulated with gluten compared to rice (*P* < .01). Subsequently, in 2015, Shahbazkhani et al. [22] performed a DBPCT on 72 adult subjects with IBS to evaluate the effect of gluten on GI symptoms. Patients were asked to follow a 6-weeks GFD during which a gluten or placebo meal was assumed daily. HLA-DQ status was also studied in all the patients. At the end of the trial statistically significant differences between the gluten containing and the gluten free groups were described regarding the symptoms control (*P* < .001), with no effect of HLA status. In 2016, Elli et al. [23] performed another DBPCT on 98 adult patients with functional GI symptoms to study the effect of gluten on satisfaction level, general well-being, and GI symptom severity, evaluated on a VAS. During the run-in phase patients had to follow a GFD for 3 weeks and, in case of a positive response, were enrolled to perform the DBPC gluten challenge, followed by a 7-days washout period before crossing over to the other treatment. During the gluten challenge, a worsening of general well-being was reported more frequently in the gluten group than in the placebo group (*P* = .05). Twenty-eight/98 (28.5%) relapsed during gluten administration, with 14/28 (50%) that were responsive also to placebo administration (nocebo effect). So, only 14/98 (14.2%) relapsed exclusively during the gluten challenge. The last DBPCT was published in 2016 by Zanwar et al. [24] who enrolled 60 adults with IBS to evaluate their clinical response to gluten administration. Overall and individual symptoms were evaluated on a VAS. All subjects had to follow a GFD for 4 weeks and, those who had an adequate response, were included in the double-blind gluten challenge after a 4-weeks washout. A greater worsening of symptoms was reported in the gluten group compared to the placebo group (*P* < .05) for both overall and individual symptoms, with the only exception of wind passage. All the details regarding the studies’ procedures are summarized in Table 2.

#### Paediatric studies

There is only one DBPC gluten challenge performed in children with chronic functional GI symptoms and published in January 2018 by Francavilla et al. [25]. In their study, the researchers screened 1114 children with functional GI symptoms diagnosed according to Rome III criteria, to evaluate the correlation between symptoms and gluten ingestion. Thirty-six out of 1114 (3.3%) subjects self-reported an association between gluten assumption and symptoms occurrence. GI symptoms’ severity was assessed by a VAS and by the IBS-SSS, while the impact on quality of life was evaluated using the State-Trait Anxiety Inventory for Children (STAIC). Five/36 (13.9%) subjects had a spontaneous improvement of symptoms during the run-in phase, before the beginning of the DBPC trial. Subsequently, 31 subjects were included in the open 2-weeks GFD, after which subjects with a significant reduction in GI symptoms were enrolled for the placebo-controlled gluten challenge. Twenty-eight children were enrolled for the gluten re-challenge, with the administration of one sachet each day of gluten (10 g/day) or placebo (rice starch) for 2 weeks, followed by the crossover to the other treatment group for other 2 weeks, after a 1-week wash-out period. At the end of the trial, the authors reported a decrease in all clinical scores during the open GFD, with 11/28 (39.2%) having a global VAS improvement > 30% between the gluten and the placebo challenge. However, no difference in the global VAS score was found during the blind administration of gluten or placebo, and 4/36 (14.3%) had an increase of symptoms during the placebo administration.

### Gluten and placebo challenge

Regarding the gluten challenge, the methods widely vary among the different studies. In fact, the specific amount of gluten administered ranges from 5.6 g/d [23] to 52 g/d [22], and sometimes is not even specified [17, 24]. Only 1 study used an unspecified amount of wheat instead of gluten [21]. Differences exist also regarding the type of placebo used in the 7 trials in which a gluten challenge was performed, including whey [18], xylose [21], rice starch alone [17, 23, 25] or combined with corn starch and glucose [22], or gluten free muffins and/or bread [16, 24]. Moreover, variability is also found in the duration of the challenge (from 1 to 6 weeks), of the washout period (from 1 to 2 weeks), and of the GFD (from 6 weeks prior the challenge to a total of 6 months). Finally, in the study by Carroccio et al. [21] the GFD is combined with an exclusion of cow’s milk, eggs, tomato, and chocolate; in the study by Biesikierski et al. [18] the GFD diet is associated with a low FODMAPs diet, while in the trial by Shahbazkhani et al. [22] the gluten meal used in the re-challenge is free of FODMAPs. Detailed information on the study procedures are summarized in Table 2.

## Discussion

In this systematic review we have included 11 studies that evaluated the clinical response to gluten in subjects with FGIDs. The risk of bias of the studies ranges from low to high, the latter mainly due to the lack of exclusion of wheat allergy in the subjects enrolled and to the blinding of participants [15–19]. Most of the studies included in this systematic review have identified a correlation between GI symptoms occurrence and gluten administration.

In recent years, the number of subjects following a GFD has increased exponentially ranging from 6.2–13% of the general population [13]. This is partly linked to the association of gluten intake with GI symptoms, and partly due to the perception of the GFD as healthier compared to a standard diet, even for people who are not affected by coeliac disease or wheat allergy. However, although the GFD is generally regarded as a safe long-term diet, it is important to note that it is not risk-free in terms of nutritional adequacy. In fact, it has been demonstrated that GFD can be associated with lower intakes of micronutrients (iron, calcium, magnesium, and vitamin D) and carbohydrates, and higher intakes of saturated fats, simple sugars and proteins, compared to the recommended daily intakes [26]. For this reason, it is important to recommend a GFD only to those subjects who can really benefit from it.

According to the prospective studies included, GFD seems to have an effect on overall GI symptoms control in subjects with FGIDs, reporting a significant decrease in GI symptoms in 29–71% of the subjects enrolled [15, 19, 20], with Whanschaffe et al. [15] describing also an improvement in stool consistency in 37% of the IBS-D subjects enrolled. However, these are prospective studies with a high risk of bias, carried on without blinding of the participants to the intervention, and without the possibility to minimize the placebo effect that is one of the major contributors to the clinical response in trials performed on subjects with IBS, with a response rate that can reach 42% [27]. Placebo effect in IBS trials seems not to be influenced by the trial duration, with evidence reporting rates of 40% of placebo response even after 52 weeks of treatment. In addition, the placebo effect in FGIDs seems to be higher in children compared to adolescents and adults [28].

Regarding As regards the gluten re-challenges, most of the studies included found a correlation between gluten ingestion and decreased control of GI symptoms in 28.5–100% of the patients evaluated. Biesiekierski et al. [16], Carroccio et al. [21] and Shahbazkhani et al. [22] described that the worsening of symptoms tends to occur within 1 week from gluten reintroduction.

Nevertheless, Biesiekierski et al. [18] failed to describe an association between gluten and GI symptoms, if FODMAPs content was simultaneously reduced in the diet, while Francavilla et al. [25] found no difference in GI symptoms control during the blind administration of gluten or placebo, reporting a worsening of symptoms in 14% of the subjects even during placebo treatment.

In 3 of the trials [16, 17, 21] the effect of gluten on bowel pattern was also evaluated, finding an improvement in patients’ satisfaction with stool consistency. Of note, Elli et al. [23] found that 28.5% of the patients relapsed during the gluten challenge, but 50% of these subjects referred a worsening of symptoms also during placebo assumption, similarly to what reported by Francavilla et al. regarding GI symptoms, in children [25]. These finding could be related to the nocebo effect that has been described in subjects with FGID, especially those referring a food intolerance, with symptoms that tend to be more present at home compared to the laboratory setting [28]. This systematic review presents some limitations. One of the major issues is related to the difficulty to compare the results of the different trials due to discrepancies in the study protocols. The first difference concerns the amount and type of gluten administered. As suggested by Catassi et al. [29], the dose of gluten for the challenge should be of 8 g/d, since this amount is not far from the average intake in the Western diet (10–15 g). However, 3 of the studies do not specify the amount of gluten administered [17, 21, 24], while 1 study uses lower doses [23]. In addition, in 3 of the trials gluten is administered as bread [24] and/or muffins [16], or as wheat [21], which makes it impossible to exclude the presence of other components that could be responsible for the symptoms. In fact, it is still not clear which component of wheat is harmful for subjects with IBS: gluten, amylase/trypsin inhibitors (ATIs), wheat germ agglutinins (WGA), or FODMAPs [13]. ATIs are wheat proteins with a protective role against parasites that can also act as triggers for the innate immunity via the Toll-like receptor 4 [30], while WGA are protective proteins contained in wheat grains that can induce the release of pro-inflammatory cytokines and alter the integrity of the intestinal epithelium, without an immune stimulatory activity [13]. Finally, FODMAPs include short chain carbohydrates that are not adsorbed in the small intestine, with subsequent fermentation and osmotic action in the large intestine [31]. As demonstrated by Biesikierski et al. in 2013 [18], a specific effect of gluten on GI symptoms was not confirmed after elimination of FODMAPs from the diet. All the abovementioned components have been associated to the occurrence of GI symptoms. The effect of FODMAPs restriction in GI symptoms’ control has been addressed in another systematic review performed on behalf of the Italian Society of Paediatrics by Turco et al. [32].

In addition, differences exist regarding the amount and duration of the gluten challenges, the type of placebo administered and the duration of the challenge itself. Moreover, in some of the trials the washout period lasts for only 1 week, that might not be sufficient to exclude the effects of previous interventions, especially in case of a cross-over design. Discrepancies exist also regarding the scoring scales used to assess GI symptoms. For this reason, it would be preferable that further studies are conducted using standardized and validated questionnaires to assess GI symptoms.

## Conclusions

In conclusion, our systematic review shows that gluten, and more probably wheat in general, can contribute to the occurrence of GI symptoms in a subgroup of patients with FGIDs, particularly in adults with IBS. However, the results of the trials available are difficult to compare due to the lack of standardization in the study designs. In children, there is only one DBPCT available and more studies are needed to draw convincing conclusions on the role of GFD for the management of GI symptoms. For this reason, currently it is not possible to recommend the use of the GFD in the routine management of FGIDs in adults and children.

## Abbreviations

AGA: Anti-gliadin
ATIs: Amylase/trypsin inhibitors
DBPCT: Double-blind placebo-controlled trials
FGIDs: Functional gastrointestinal disorders
FODMAPs: Fermentable oligo-di-mono-saccharides and polyols
GFD: Gluten free diet
GI: Gastrointestinal
IBS: Irritable bowel syndrome
IBS-SSS: IBS-Symptom Severity Score
MFH: Multiple food hypersensitivity
NCGS: Non-coeliac gluten sensitivity
NCWS: Non-coeliac wheat sensitivity
PBMCs: Peripheral blood mononuclear cells’
VAS: Visual analog scale
WGA: Wheat germ agglutinins
WS: Wheat sensitivity
